# Low-volume resistance training: a feasible, cost-effective strategy for musculoskeletal frailty in older adults attending daycare centers

**DOI:** 10.3389/fspor.2025.1542188

**Published:** 2025-04-08

**Authors:** Frederico Abreu, André Rodrigues, Fátima Baptista

**Affiliations:** ^1^ Department of Sports and Health, CIPER, Faculdade de Motricidade Humana, Universidade de Lisboa, Lisbon, Portugal; ^2^ Medical Department, Emeis Portugal, Lisbon, Portugal

**Keywords:** resistance training, older adults, falls, frailty, sarcopenia

## Abstract

**Introduction:**

Frailty is a prevalent geriatric syndrome, posing significant health risks for older adults attending daycare centers or residing in institutional settings. Addressing frailty with interventions that are feasible and cost effective and also promote high adherence within these environments is crucial.

**Objective:**

This study aimed to evaluate the impact of a low-volume, remotely supervised resistance training protocol on physical frailty among frail older adults attending daycare centers. Secondary outcomes included changes in sarcopenia prevalence and fall risk.

**Methods:**

Thirty-one frail older adults participated in a 12-week usual care period, followed by a 12-weeks intervention featuring low-volume (10-minute sessions) resistance training three times weekly. The program was delivered locally by non-specialized staff under remote supervision. Musculoskeletal, functional, and clinical assessments were conducted at three-time points: baseline, pre-intervention, and post-intervention.

**Results:**

During the usual care period, a decline was observed in handgrip strength (19.2–18.5 kg) and sit-to-stand time (14.5–17.4s) (*p* < 0.05). However, these measures were preserved during the intervention. Relative muscle power decreased during the usual care but improved with training (4.3–5.2 W/Kg, *p* < 0.001). While body composition, physical function, gait speed, and Short Performance Physical Battery scores remained stable, reductions were observed in exhaustion and physical inactivity prevalence (*p* < 0.05). Frailty prevalence showed a decreasing trend (48%–26%, *p* = 0.099), with significant reductions in sarcopenia prevalence (29%–10%, *p* = 0.045), and fall frequency (*p* = 0.022).

**Conclusion:**

The low-volume strength training protocol was a feasible, cost-effective strategy for mitigating musculoskeletal frailty criteria, sarcopenia and fall risk among older adults in daycare centers, potentially delaying the progression of these conditions.

## Introduction

Frailty is a multifaceted geriatric syndrome characterized by reduced physiological reserves and heightened vulnerability to stressors (1). This condition results in diminished strength, aerobic capacity and physical function (2). With a high prevalence among institutionalized older adults and those attending daycare centers (3–5), adverse outcomes such as loss of quality of life and independence (6), increased healthcare costs (7), and premature mortality (8) are real concerns for caregivers. Although standardizing criteria and algorithms for diagnosing frailty remains challenging (3, 9), Fried's physical frailty phenotype (1) has become a widely accepted approach. This phenotype identifies frailty based on at least three of five criteria: muscle weakness, gait slowness, low physical activity, unintentional weight loss and exhaustion.

Given the substantial neuromuscular involvement in frailty (10, 11), resistance training has emerged as a cornerstone for prevention and management (12–15). Evidence indicates that resistance training (16, 17) can improve gait speed (18), muscle strength (19) and muscle mass (20, 21). Consequently, resistance training is recommended as first-line therapy for frailty management as part of multicomponent physical activity programs, as the International Clinical Practice Guidelines for Sarcopenia (ICFSR) (22) emphasize. Additionally, frailty often overlaps and co-occurs with sarcopenia (23–25)—an age-related disease characterized by the progressive loss of muscle mass and function (26)—and the risk of falls (27), a major cause of death and disability in older adults. These geriatric syndromes share disability pathways as well as clinical and functional outcomes. Therefore, given the typical neuromuscular decline, a program focusing on frailty management could also target these conditions (28, 29).

Despite its well-documented benefits, structured resistance training programs tailored to frail older adults remain rare in institutional and daycare settings (28). Barriers include institutional limitations, such as insufficient resources and trained staff, and individual challenges, including low adherence due to fatigue or functional limitations (30). Addressing these barriers requires innovative approaches, such as low-cost, low-time-demand programs that can be implemented without significantly disrupting participants' daily routines. Furthermore, research highlights the importance of tailoring exercise interventions to participants' needs, including minimal-dose protocols that still achieve meaningful clinical benefits (31, 32). Several proposed resistance exercise programs have been based on the dose-response relationship for healthy older adults (33), which might disregard specific characteristics of frail individuals (34) (e.g., low adherence, reduced energy and exercise tolerance). Therefore, planning programs adapted to participants' abilities is essential, focusing on low-volume yet highly effective approaches (35, 36). The conception of how much resistance training is enough has not yet been clarified (35, 37), but investigating minimal dose methodologies is a vital step towards defining ideal protocols for managing and treating geriatric syndromes.

The present study evaluated the feasibility and effectiveness of a low-volume, remotely supervised resistance training program for frail older adults attending daycare centers. The program was tailored to institutional constraints and participant capabilities, addressing frailty while assessing secondary outcomes such as sarcopenia and fall prevalence. By exploring a minimal-dose approach, this study seeks to inform the development of scalable exercise protocols for managing frailty and related geriatric syndromes.

## Materials and methods

### Study design and participants

This study recruited 62 participants from four Portuguese daycare centers. Before the intervention began, one center withdrew, and several participants were unable to continue, living a final cohort of 44 individuals. Participants were required to be 65 years or older, classified as frail or pre-frail, and physically and cognitively able to follow basic exercise instructions. A physician provided medical clearance for all participants.

The study employs a repeated measures design, with each participant undergoing a 12-week usual care phase (control) followed by a 12-week exercise intervention. Assessments were conducted at three-time points: baseline (T_I_), pre-intervention (T_II_), and post-intervention (T_III_).

### Musculoskeletal evaluation

Musculoskeletal fitness was assessed through handgrip strength (Leonardo Mechanography, Novotec Medical, Pforzheim, Germany) and appendicular skeletal muscle mass (ASMM) measured via bioimpedance (Bio 101, Akern, Wurzburg, Germany). After a brief explanation and familiarization with the device, maximal handgrip strength was measured twice, with participants seated on a 45 cm high armless chair and the dominant arm's elbow flexed at 90°. The highest value was recorded. ASMM assessment was conducted five minutes after participants were immobilized in a lying position, with the skin prepared and bioimpedance electrodes placed. Resistance and reactance were recorded, converted to ASMM (38), and adjusted for body mass.

### Functional evaluation

Physical function was assessed using the Short Physical Performance Battery (39) (SPPB), which includes gait speed, standing balance, and a five times sit-to-stand test (5STS). Gait speed was measured during two attempts of a 2.44 m walk at the participant's usual pace. Due to potential alterations in gait fluency, which are common among older adults, the fastest attempt was recorded for analysis. Balance was assessed using three progressively challenging foot placements: parallel, semi-tandem, and full tandem. Participants were required to maintain each position for up to 10 s. The test was concluded for any participant unable to sustain balance for the full duration in any position. Lower-limb strength was assessed with the 5STS test, which measured the time required for participants to rise from a seated position (45 cm high) five times without using their hands. The test was performed on a force reaction platform (Leonardo Mechanography, Novotec Medical, Pforzheim, Germany) that recorded both the completion time and muscle power generated during movements.

The three physical function tests (gait speed, balance and lower-limb strength) were scored from 0–4, resulting in a maximum total of 12 points on the SPPB Scale, with higher scores representing better physical function. Participants who were unable to stand or walk independently received a score of 0 on the respective SPPB test and were excluded from the statistical analysis of gait speed.

In addition, the perceived capacity to perform activities of daily living (ADL) was assessed using the 12-item Composite Physical Function (40) (CPF) scale. This self-reported questionnaire evaluates physical function across three domains: basic, instrumental, and advanced ADLs. Each of the 12 items was scored from 0–2, with a maximum possible score of 24 indicating high physical function. The caregiving staff also reported the number of falls experienced by each participant over the previous 12 weeks.

### Clinical evaluation

Frailty was diagnosed using Fried's phenotype (1), which includes five criteria: muscle weakness, unintentional weight loss, gait slowness, low physical activity, and self-reported exhaustion. Muscle weakness was determined by handgrip strength thresholds based on sex and body mass index. Unintentional weight loss was defined as a loss exceeding 5 kg or 5% of body mass within the past 12 months. Gait slowness was identified if walking velocity fell below 0.6 or 0.7 m/s, depending on sex and height. Self-reported exhaustion was assessed using the two questions from the Center of Epidemiology Scale for Depression (CES-D) questionnaire while low physical activity was evaluated based on daily mobility with International Physical Activity Questionnaire—Short Form (41, 42). Participants meeting one or two of these criteria were classified as prefrail while those meeting three or more were categorized as frail.

Sarcopenia was assessed according to the EWGSWOP 2019 algorithm (26). Dynapenia was identified if handgrip strength was ≤27 kg in men and 16 kg in women. Sarcopenia was confirmed if appendicular skeletal muscle mass index (ASMMI), calculated as ASMM divided by height squared, was ≤7 kg/m^2^ in men and ≤5, 5 kg/m^2^ in women.

### Physical exercise training protocol

All participants followed a low-volume resistance exercise program conducted without specialized supervision. To enable autonomous delivery of the program, two formal training sessions were provided to caregiving staff at the daycare centers, including occupational therapists and sociocultural animation professionals. This approach addressed the limitation of needing an exercise physiologist for training sessions at each institution. Although the caregiving staff were not exercise specialists, they were experienced in leading various activities and were familiar with the specific care needs of frail older adults.

Each exercise session included two sets of three different resistance band (35) exercises, with 6–10 repetitions per set and 90 s rest intervals between sets and exercises. Sessions were designed to last no more than 15 min and were conducted thrice weekly, with a minimum 48 h rest period between sessions. Participants were encouraged to perform each set to volitional fatigue, which was defined as the point at which they either reported an inability to continue or were unable to maintain proper technique, emphasizing an explosive concentric phase followed by a slower eccentric phase. Supervisors were instructed to adjust the elastic band resistance if volitional fatigue was not reached during a given exercise. This was achieved by replacing the band with one of higher tension (three tension levels were available). If the participants already used the highest-tension band, their grip position was adjusted by shortening the distance to the elastic band's fixed point by approximately 20 centimeters.

Once training routines were established, staff reported that participants usually did not reach high fatigue levels and tended to rest for smaller periods, which meant that session time did not surpass 10 min. The program comprised nine different exercises distributed unevenly across the 12-week intervention. The primary exercises—chest press, squat, and seated rows—were prioritized, averaging 3.3 ± 1.6 sets per week, as they were considered critical for achieving the study's primary outcomes (36). Secondary exercises (leg extension, seated hip abduction, seated hip flexion, side raises, elbow flexion, and seated reverse flies), were performed less frequently, with an average of 1.2 ± 0.6 sets per week.

A pre-recorded video displayed on a large screen demonstrating the correct form for each exercise, ensured participants could easily follow and perform the routines as intended. Exercise sessions were conducted in groups of up to eight participants per supervising caregiving staff member. To qualify for inclusion in the study, participants were required to attend at least 31 sessions, representing an adherence rate of over 85%.

Initially designed as a low-volume resistance training protocol (36), the program can also be classified as minimum-dose power training (17), due to its focus on rapid concentric movements and the avoidance of high muscle fatigue levels during exercise.

### Statistical analysis

All statistical analyses were performed using the SPSS statistics software package (version 28.0 for Mac; IBM, Chicago, IL, USA). The analysis comprised descriptive statistics (mean and standard deviation) to characterize the sample's anthropometric (height, weight and body mass index), neuromuscular (ASMM, handgrip strength, 5STS time and muscle power), and functional (gait speed, SPPB, CPF and number of falls) variables. The prevalence of frailty, sarcopenia, falls, and related criteria (weight loss, muscle weakness, gait slowness, low physical activity and exhaustion) was also determined at each time point.

A repeated-measures ANOVA was used to evaluate the effects of the control and intervention periods on continuous variables (anthropometric, neuromuscular and functional). Mauchly's test was performed to assess whether the sphericity assumption had been met. When the assumption was violated, Greenhouse-Geisser or Huynh-Feldt corrections were applied to adjust the degrees of freedom. *Post-hoc* pairwise comparisons using the Bonferroni correction were performed if significant results were found in the repeated-measures ANOVA.

For categorical variables, including the prevalence of syndromes and positive frailty or sarcopenia criteria, a Cochran's *Q* Test was employed to detect significant differences between time points during the control and intervention periods. If significant differences were observed, *post-hoc* pairwise comparisons with Bonferroni corrections were conducted to identify specific time-point differences. A *p*-value of less than 0.05 was considered statistically significant for all analyses.

## Results

Of the 44 participants who began the 12-week exercise intervention, 31 (61% females) completed it. Dropouts were primarily due to participants changing daycare centers (*n* = 11), falls requiring surgery (*n* = 1), and severe pre-existing illness (*n* = 1). Despite the lower completion rate, those who remained in the program demonstrated high adherence to the exercise sessions, with attendance exceeding 90% across all participants. No participants voluntarily withdrew from the study, and only minimal adverse effects, such as mild muscle soreness during initial sessions, were observed, highlighting the program's safety for this population. All daycare centers that initiated the exercise program continued until the end of the 12 weeks, demonstrating its feasibility and adaptability within institutional settings.

At the baseline (T_I_), participants had an average age of 82.29 years, with 38.7% classified as frail, 22.6% diagnosed with sarcopenia, and 35.5% reporting a recent fall. Gait slowness and muscle weakness emerged as the most prevalent frailty criteria throughout the 24-weeks study period.

**Figure 1:**
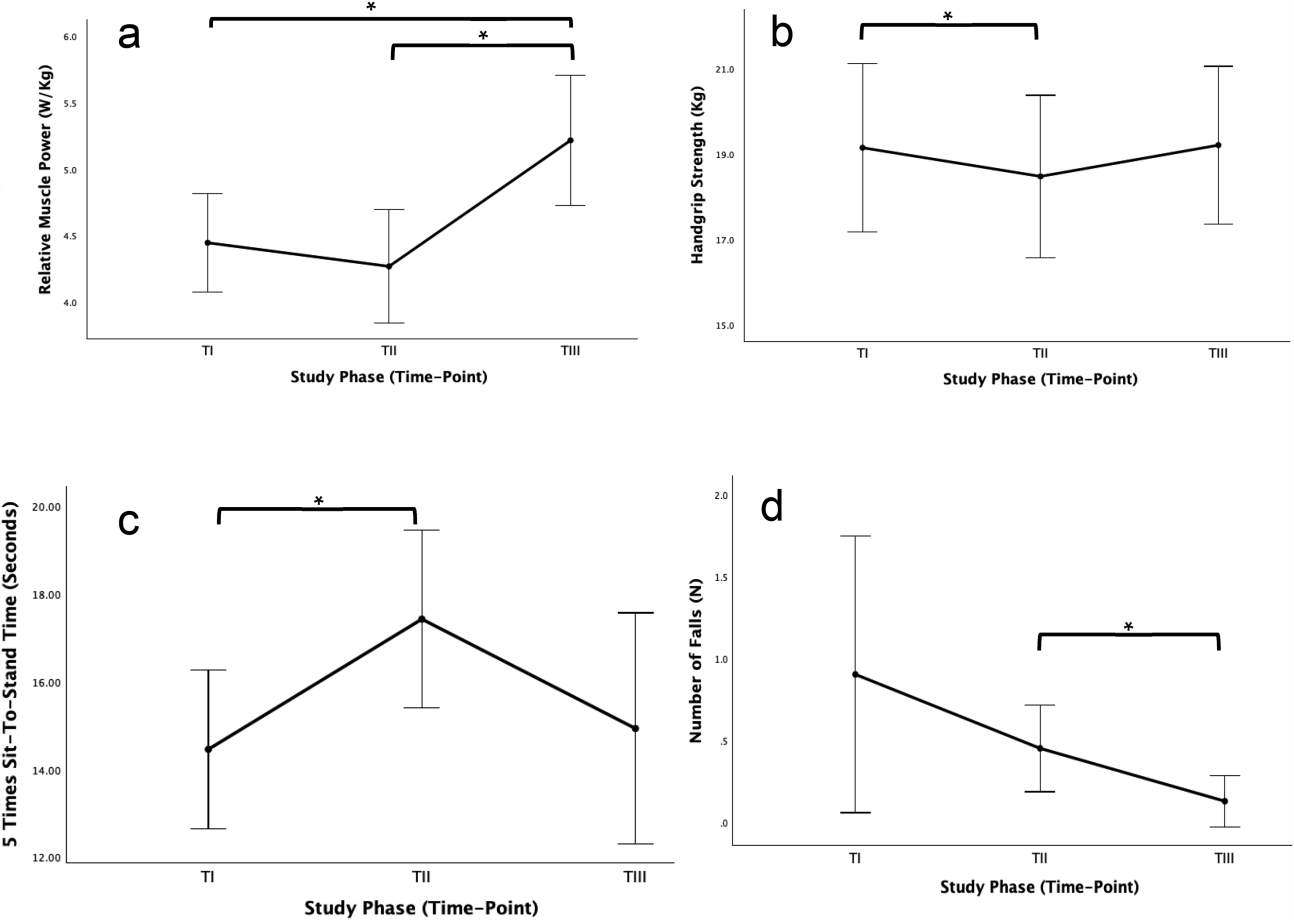
Effect of physical exercise intervention in relative muscle power **(a)**, handgrip strength **(b)**, 5 times sit-to-stand time **(c)** and number of falls **(d).**

Neuromuscular function declined significantly from T_I_ to T_II_, particularly handgrip strength (Figure 1b), 5STS time (Figure 1c), and absolute and relative muscle power (Table 1). However, this trend was markedly reversed during the intervention period (T_II_ to T_III_), with significant improvements observed in muscle power (Figure 1a). Nevertheless, no statistically significant effects of the intervention or the usual care period were found on anthropometric measures (BMI and ASMM) or physical function variables (SPPB, CPF and gait speed).

**Table 1:** Effects of exercise intervention in neuromuscular and functional parameters (Mean ± SD).

Variables	T_I_	T_II_	T_III_	>	*p*-value
BMI, kg/m^2^	28.53 ± 5.40	28.32 ± 5.45	28.73 ± 5.20	-	0.204
ASMM, kg	17.48 ± 3.27	17.21 ± 3.19	17.03 ± 3.22	-	0.322
ASMMI, kg/m^2^	7.15 ± 1.11	7.04 ± 1.10	6.96 ± 1.15	-	0.339
Gait Speed, m/s	0.65 ± 0.28	0.59 ± 0.21	0.62 ± 0.25	-	0.256
SPPB, pts	7.61 ± 2.26	7.42 ± 2.53	7.77 ± 2.82	-	0.609
CPF, pts	13.00 ± 6.75	13.30 ± 6.36	13.73 ± 5.63	-	0.545
Muscle Power, kw	0.31 ± 0.08	0.29 ± 0.08	0.36 ± 0.09	T_III_ > T_I_, T_II_	<0.001*
Relative Muscle Power, W/kg	4.45 ± 1.01	4.27 ± 1.16	5.21 ± 1.34	T_III_ > T_I_, T_II_	<0.001*
Grip Strength, kg	19.15 ± 5.37	18.48 ± 5.18	19.21 ± 5.03	T_I_ > T_II_	0.107*
5x Sit-to-Stand, s	14.45 ± 4.92	17.42 ± 5.51	14.93 ± 7.18	T_II_ > T_I_	0.053*
Number of Falls, n	0.90 ± 2.30	0.45 ± 0.72	0.13 ± 0.43	T_II_ > T_III_	0.138*

T_I_, baseline; T_II_, pre-intervention; T_III_, post-intervention; >, study phase comparison when significant differences were found; *p*-value, significance for the repeated-measures ANOVA; BMI, body mass index; ASSM, appendicular skeletal muscle mass; ASSMI, appendicular skeletal muscle mass index; SPPB, short performance physical battery; CPF, composite physical function; *, indicates significant differences between study phases.

Apart from muscle weakness, the prevalence of all frailty criteria tended to decrease from T_II_ to T_III_, with the most significant reductions observed in low physical activity and exhaustion (Table 2). While the percentage of participants below the threshold for gait slowness increased from T_I_ to T_II_ (61.3%–71.0%) and subsequently decreased from T_II_ to T_III_ (71.0%–58.3%), these changes were not statistically significant. However, changes in neuromuscular and behavioral variables contributed to a clear trend of reduced frailty prevalence, which dropped from 48.4% at T_II_ to 25.8% at T_III_.

**Table 2:** Effects of exercise intervention in syndrome prevalence.

Syndromes	T_I_	T_II_	T_III_	>	*p*-value
Frailty Criteria					
Gait Slowness, %	61.3	71.0	58.1	-	0.273
Weakness, %	87.1	90.3	93.5	-	0.368
Low PA, %	32.3	25.8	0.0	T_I_, T_II_ > T_III_	<0.001*
Exhaustion, %	22.6	41.9	25.8	T_I_, T_II_ > T_III_	0.012*
Weightloss, %	16.1	16.1	9.7	-	0.670
Frailty, %	38.7	48.4	25.8	-	0.099
Sarcopenia Algorithm					
Upper-Limb Low Muscle Strength, %	64.5	74.2	48.4	T_I_, T_II_ > T_III_	0.004*
Lower-Limb Low Muscle Strength, %	51.6	67.7	29.0	T_I_, T_II_ > T_III_	0.001*
Low ASMMI, %	25.8	32.3	25.8	-	0.607
Low ASMM, %	41.9	48.4	58.1	T_III_ > T_I_	0.042*
Low Physical Performance, %	64.5	64.5	54.8	-	0.500
Sarcopenia, %	22.6	29.0	9.7		0.045
Fallers, %	35.5	32.3	9.7	T_I,_ T_II_ > T_III_	0.022*

T_I_, baseline; T_II_, pre-intervention; T_III_, post-intervention; >, study phase comparison when significant differences were found; *p*-value, significance for the Cochran's *Q* Test; PA, physical activity; ASSM, appendicular skeletal muscle mass; ASSMI, appendicular skeletal muscle mass index; *, indicates significant differences between study phases.

**Figure 2:**
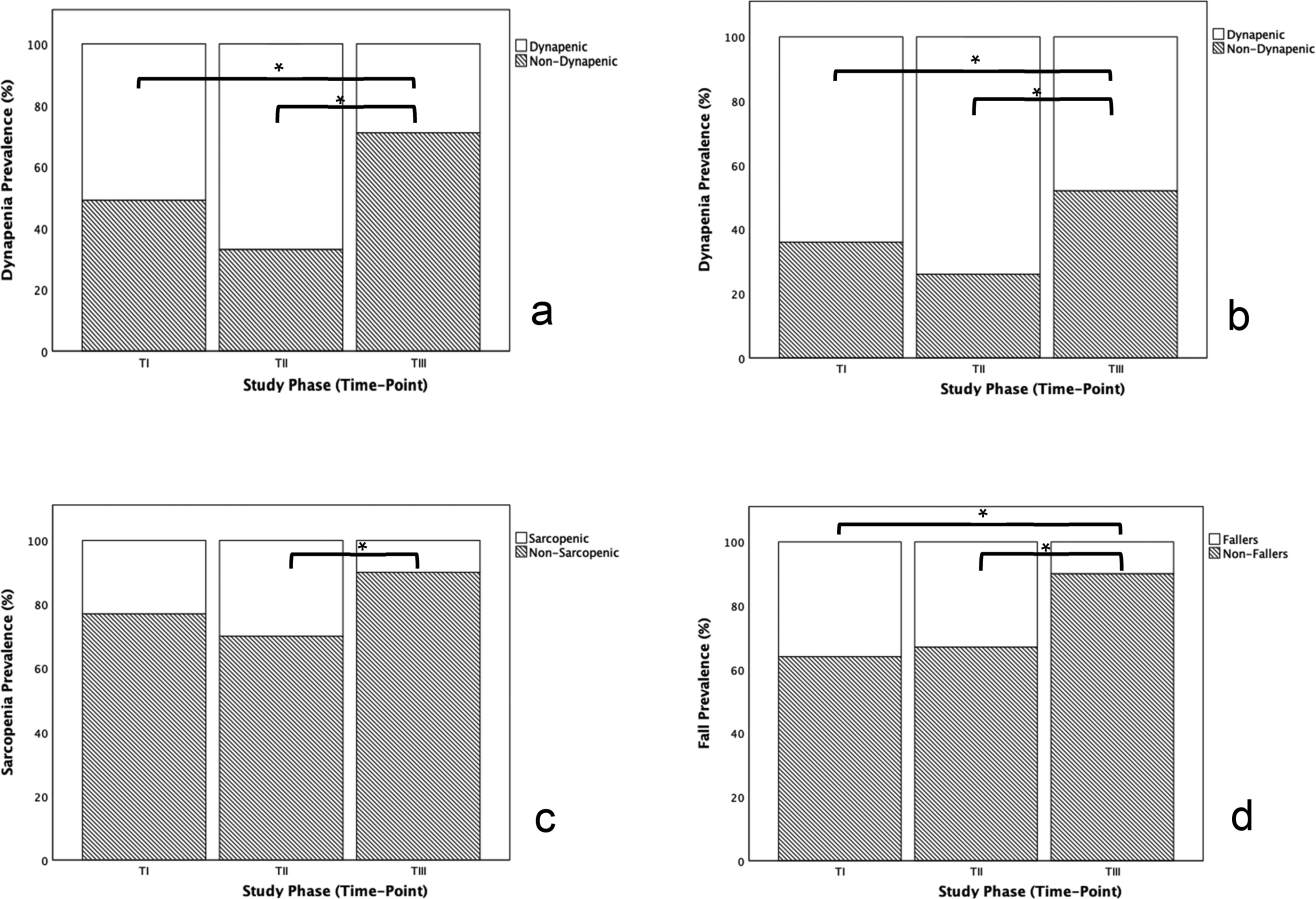
Effect of physical exercise intervention in the prevalence of dynapenia assessed in the upper limb **(a)**, dynapenia assessed in the lower limb **(b)**, sarcopenia **(c)** and falls **(d).**

The proportion of participants falling below the muscle strength threshold for sarcopenia decreased significantly from T_II_ to T_III_ (Figures 2a,b), irrespective of the assessment site (upper or lower limb). According to the applied algorithm, this resulted in a reduction in sarcopenia prevalence (*p* = 0.014), even in the absence of measurable increases in muscle mass (Figure 2c). Additionally, the total number of falls (Figure 1d), particularly the percentage of fallers (Figure 2d), decreased during the intervention, dropping from over 30% at T_I_ and T_II_ to just 9.7% at T_III_.

## Discussion

The present study investigated the impact of a low-volume, remotely supervised resistance training program on frailty criteria among frail older adults attending daycare centers. The findings demonstrate that this approach can effectively prevent decline or promote improvements in key physical frailty indicators, potentially reversing or delaying its progression.

During the usual care period, significant declines were observed in handgrip strength and 5STS time, both crucial parameters of neuromuscular function in older adults. Although the loss of muscle strength and function was anticipated (43, 44), the rate of deterioration was notable given the relatively short duration of the usual care phase. In line with current knowledge (20, 45), the implementation of the resistance training program successfully halted this decline, effectively preserving muscle capacity. Furthermore, muscle power improved by 22% during the intervention period. While previous research has linked improvements in muscle power to enhanced gait speed, evidence suggests that a minimum 30% increase in muscle power may be required to impact gait speed significantly (46). Although resistance training is known to strongly affect gait speed (18), our results did not show significant changes in this variable, possibly due to a type II statistical error. This is supported by the observed trend: a decline in gait speed during the usual care phase, followed by a slight improvement during the intervention.

The absence of significant changes in objective physical function (SPPB) or perception of capacity (CPF) may reflect the intervention's duration and low volume, which may have been insufficient to elicit detectable adaptations. Previous studies have shown that a minimum of 8.2% improvement in one-repetition maximum (1RM) strength is required for significant increases in physical function, a measure that is challenging to achieve with resistance band exercises (46).

Variations in physical function and neuromuscular capacity following the intervention translated into mixed effects on specific frailty criteria. Although the prevalence of gait slowness showed a slight but statistically insignificant reduction, there were no substantial changes in the prevalence of muscle weakness or unintentional weight loss. Importantly, however, significant reductions were observed in the prevalence of exhaustion and low physical activity, both of which are perceptive, self-reported measures. These changes likely reflect participants' subjective sense of increased energy and greater engagement in daily activities rather than objectively measured physical improvements. Previous research has consistently shown that resistance training alone does not improve habitual physical activity (47, 48). Nevertheless, the increase in physical activity observed in this study was sufficient to bring all participants above the low physical activity criterion threshold.

Overall, these changes contributed to a tendency towards a decrease in frailty prevalence, aligning with previous findings (13, 19, 49, 50) that support resistance training as a therapeutic tool for frailty management.

Supervision has consistently been shown to enhance outcomes in exercise programs for older adults (51, 52) and to improve adherence (53). This study represents the first attempt to implement a remotely supervised exercise program at an institutional level for this population. The findings suggest that unspecialized local supervision is sufficient to drive meaningful changes in physical function and frailty-related measures. Nevertheless, direct specialized supervision may have yielded even greater improvements emphasizing the potential for hybrid supervision models that combine remote and on-site expertise. On the other hand, the program's design achieves full adherence by participants and institutions, addressing common barriers to implementation (30, 32), enabling consistent participation.

The intervention had no measurable effect on ASMM, despite resistance training being recognized as a primary intervention for increasing muscle mass (45). High-intensity effort is typically necessary to stimulate muscle growth (20) and this program's intensity may not have met that threshold (35). Additionally, nutritional support is often needed to optimize muscle hypertrophy outcomes (54, 55). The limitations of bioimpedance analysis, a tool with known constraints, may have further hindered the accurate detection of changes in muscle mass (56). Despite the lack of significant muscle mass or strength gains, the observed improvement trend in handgrip strength and the 5STS time which progressed, was sufficient to reduce the prevalence of dynapenia following resistance training. According to the EWGSWOP2 (26) algorithm, this represented a substantial decline in sarcopenia prevalence. Notably, different thresholds for low muscle strength in diagnosing sarcopenia (26) and frailty (1) led to significantly divergent results.

Finally, the observed reduction in fall frequency and the percentage of fallers is a critical finding, given the severe consequences of falls in older adults. This finding is consistent with previous studies demonstrating the efficacy of resistance training in improving balance and reducing fall incidence (57). However, the magnitude of this reduction is especially noteworthy.

Our results indicate that even a low-volume resistance training program can yield meaningful benefits for managing frailty, sarcopenia and fall prevalence. However, the study has limitations. The repeated measures design suggests that participants may have experienced physical decline before the intervention began, potentially complicating comparisons between the control and intervention periods. The use of resistance bands, while enhancing adherence and addressing institutional constraints, limited the ability to precisely control exercise intensity, progression, and task-specific muscle strength assessment. Furthermore, the absence of nutritional support and more robust tools for measuring muscle hypertrophy likely affected the evaluation of muscle mass changes.

Future research should focus on developing feasible exercise programs tailored to individual and institutional needs. Studies exploring optimal volumes and frequencies in larger samples and over longer durations are essential to understand better how older adults adapt to low-volume resistance training protocols.

## Conclusion

This study highlights the benefits of a low-volume, remotely supervised resistance training program for frail older adults attending daycare centers. The findings demonstrate that such a program can overcome common institutional and individual barriers to implementing exercise interventions in senior care settings. Despite its limitations, this study underscores the potential of this approach to improve outcomes related frailty, sarcopenia, and falls. Further research is needed to refine the program design balancing individual and institutional adherence with meaningful clinical improvements.

## Data Availability

The raw data supporting the conclusions of this article will be made available by the authors, without undue reservation.
